# Mobile Health Technology (mDiab) for the Prevention of Type 2 Diabetes: Protocol for a Randomized Controlled Trial

**DOI:** 10.2196/resprot.8644

**Published:** 2017-12-12

**Authors:** Shruti Muralidharan, Viswanathan Mohan, Ranjit Mohan Anjana, Sidhant Jena, Nikhil Tandon, Steven Allender, Harish Ranjani

**Affiliations:** ^1^ Global Obesity Centre World Health Organization Collaborating Centre for Obesity Prevention Deakin University Geelong Australia; ^2^ Translational Research Department Madras Diabetes Research Foundation Dr. Mohan's Diabetes Specialities Centre Chennai India; ^3^ Janacare Solutions Private Limited Bengaluru India; ^4^ Department of Endocrinology All India Institute of Medical Sciences New Delhi India

**Keywords:** prevention, diabetes mellitus, type 2, mHealth

## Abstract

**Background:**

The prevalence of type 2 diabetes is increasing in epidemic proportions in low- and middle-income countries. There is an urgent need for novel methods to tackle the increasing incidence of diabetes. The ubiquity of mobile phone use and access to Internet makes mobile health (mHealth) technology a viable tool to prevent and manage diabetes.

**Objective:**

The objective of this randomized controlled trial is to implement and evaluate the feasibility, cost-effectiveness, and sustainability of a reality television–based lifestyle intervention program. This intervention program is delivered via a mobile phone app (mDiab) to approximately 1500 Android smartphone users who are adults at a high risk for type 2 diabetes from three cities in India, namely, Chennai, Bengaluru, and New Delhi.

**Methods:**

The mDiab intervention would be delivered via a mobile phone app along with weekly coach calls for 12 weeks. Each participant will go through a maintenance phase of 6 to 8 months post intervention. Overall, there would be 3 testing time points in the study: baseline, post intervention, and the end of follow-up. The app will enable individuals to track their weight, physical activity, and diet alongside weekly video lessons on type 2 diabetes prevention.

**Results:**

The study outcomes are weight loss (primary measure of effectiveness); improvement in cardiometabolic risk factors (ie, waist circumference, blood pressure, glucose, insulin, and lipids); and improvement in physical activity, quality of life, and dietary habits. Sustainability will be assessed through focus group discussions.

**Conclusions:**

If successful, mDiab can be used as a model for translational and implementation research in the use of mHealth technology for diabetes prevention and may be further expanded for the prevention of other noncommunicable diseases such as hypertension and cardiovascular diseases.

**Trial Registration:**

Clinical Trials Registry of India CTRI/2015/07/006011 http://ctri.nic.in/Clinicaltrials/pdf_generate.php? trialid=11841 (Archived by WebCite at http://www.webcitation.org/6urCS5kMB)

## Introduction

The International Diabetes Federation estimates the global prevalence of type 2 diabetes mellitus (T2DM) to rise to 642 million by 2040 from the current number of 415 million [1]. Three-fourths of this population lives in the low- and middle-income countries (LMICs) such as India [1-3]. Studies in different populations have shown that a large part of T2DM is attributed to factors such as obesity (both generalized and abdominal), sedentary lifestyle, and poor diet [4-7]. Anjana et al [8] assessed the population attributable risks in an urban Indian population and reported that by modifying just two factors, namely, diet and physical activity, up to 50% of new onset T2DM can be prevented.

Several randomized controlled trials (RCTs) carried out on primary prevention of T2DM have shown that lifestyle modification and metformin therapy can reduce the incidence of T2DM in people who are in the prediabetes stage, that is, those having impaired glucose tolerance (IGT) or impaired fasting glucose (IFG), or both [9-16]. However, most of these trials were conducted face to face. This involves a lot of man power, cost, and time. The question arises whether similar results can be obtained using modern technology without the need for face-to-face interaction.

The World Health Organization (WHO) considers mobile health (mHealth) as a component of electronic health (eHealth). The definition of eHealth according to the WHO is “the use of information and communication technologies for health,” and mHealth is defined as “medical and public health practice supported by mobile devices, such as mobile phones, patient monitoring devices, personal digital assistants (PDAs), and other wireless devices” [17].

It is of interest to note that India stands second worldwide both in the prevalence of type 2 diabetes [1] as well as in the number of mobile phone users [18]. According to the statistics provided by the Telecom Regulatory Authority of India, the number of wireless telephone users in India increased from around 1034 million in April 2016 to around 1174 million in January 2017, that is, 100 million users added within a year [19]. Moreover, the Internet and Mobile Association of India (IAMAI) and Indian Market Research Bureau (IMRB) statistics have also shown that India has the second largest population using the Internet, of which a large number gain access to the Internet using their smartphones. The IAMAI and IMRB prediction for the mobile-based Internet users is that there would be 371 million users by June 2017 from 306 million in December 2016, which represents an increase of 21% in just 6 months [20]. This massive upswing and uptake of technology in the field of mobile technology renders mHealth as a liable opportunity to provide individual-level support beyond traditional clinic-based care technology [17,20-24].

With this background information, we took up a trial of primary prevention of diabetes based on mHealth. The aims of this trial are to understand the feasibility, cost-effectiveness, and sustainability of a reality television–based lifestyle intervention delivered via a mobile phone app along with the support of a health coach for 12 weeks and to evaluate its effect on weight loss, cardiometabolic risk factors, and behavioral and social variables, such as physical activity, quality of life (QOL), and adoption of healthier diet and lifestyle behaviors, in Android mobile phone users who are at a high risk of developing T2DM.

## Methods

### Study Design and Participants

**Figure 1:**
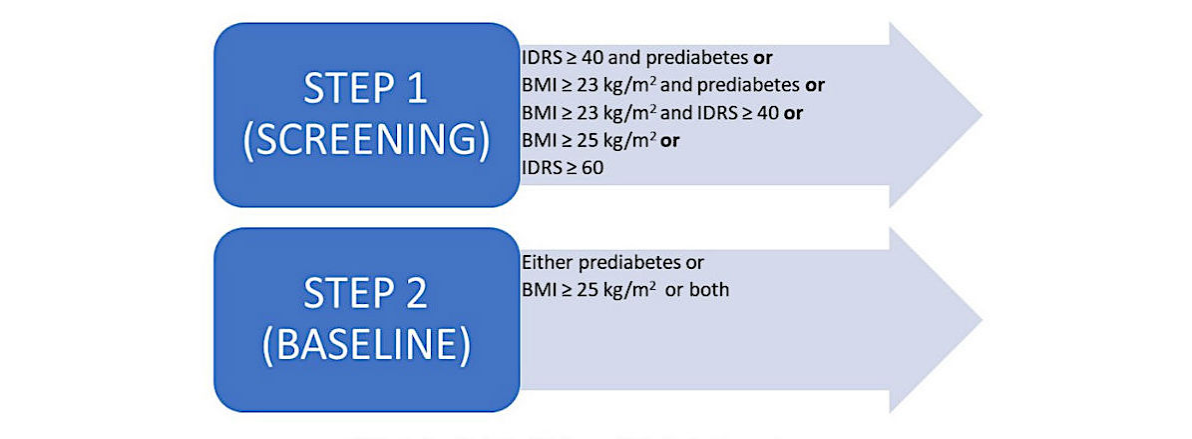
Eligibility criteria at screening and baseline. IDRS: Indian Diabetes Risk Score; BMI: body mass index.

This study is an RCT being conducted at three cities in India (Chennai, Bengaluru, and New Delhi) between 2016 and 2018. Android mobile phone users, between the age groups of 20 and 65 years, are screened for eligibility based on a combination of screening criteria detailed in Figure 1. The rationale for choosing Android users is based on the data published in 2016 that 97% of the mobile phone users in India use the Android operating system [25]. This figure rose from 90% in 2015 [25].

### Eligibility Criteria

The screening criteria are designed to ensure that individuals at a high risk of developing diabetes are selected for the study. To select the high-risk group, a combination of the Indian Diabetes Risk Score (IDRS), body mass index (BMI), and capillary blood glucose (CBG) is used.

IDRS is a simplified and validated tool that uses 4 parameters, namely, age, abdominal obesity, family history of diabetes, and physical activity [26,27]. It was shown that a high IDRS score increases with the risk of T2DM, metabolic syndrome, and cardiovascular disease [28]. Generally, an IDRS score of 40 indicates a moderate risk of developing diabetes and a score of 60 indicates a high risk. For the purpose of including a wide range of high-risk population, we classified an IDRS score of ≥40, along with either a BMI indicative of obesity or CBG indicative of prediabetes (IFG=100-125 mg/dL or IGT=110-199 mg/dL) to determine eligibility at screening. However, an IDRS score of ≥60 is considered as an independent criterion to determine eligibility. BMI is an important diagnostic marker for overweight and obesity [29]. We set a cut point of ≥23 kg/m^2^to indicate overweight and obesity as defined by the WHO Asia Pacific guidelines [30], and this is used in combination with CBG, indicating prediabetes and/or and an IDRS ≥40. However, a BMI ≥25 kg/m^2^ is considered as an independent criterion to determine eligibility. The CBG testing is used to identify an IFG of 100 to 125 mg/dL or an IGT of 110 to 199 mg/dL at screening using the WHO cut-offs [31]. The final eligibility criterion for entry into the trial is defined as either an IFG of 100 to 125 mg/dL and/or an IGT of 140 to 199 mg/dL through a 75-g oral glucose tolerance test and/or a BMI ≥25 kg/m^2^, indicating obesity at baseline (Figure 1).

The exclusion criteria for the study are participants who are pregnant or breastfeeding, are involved in other wellness programs, whether they have been previously diagnosed with diabetes or have a history or evidence of heart disease or any other serious illness, are children or young people (ie, aged <20 years) or older adults (ie, aged >65 years), or have conditions (such as orthopedic conditions) that would impede participation in this study.

### Recruitment

Recruitment is carried out through screening camps at clinic settings, corporates/worksites, residential colonies/gated housing complexes, educational institutions, religious or spiritual institutes, open public spaces, and direct referrals (such as participants referred by study staff and physicians) to cover the general population at large.

If an individual fulfilled 1 or more of the criteria enlisted in Step 1 (Figure 1), they are invited for baseline testing, and each participant is subjected to a panel of tests as detailed in Table 1 to ensure final eligibility into the study (Step 2 in Figure 1). At each site, approximately 3000 people are screened based on the screening criteria shown in Figure 1, and approximately 1000 individuals would be available for baseline testing. The main aim of the baseline visit is to ensure that the individuals screened do not have type 2 diabetes [31] or fall under any other exclusion criteria and that they have understood the requirements of the intervention study. A summary of measures that are conducted at each visit are detailed in Table 1 and Multimedia Appendix 1.

**Table 1:** Summary of study measures.

Measurements		Screening	Baseline	Post intervention	End of follow-up
**Questionnaires**					
	Short questionnaire	✓			
	Questionnaires measuring participants’ health habits, diet behavior, quality of life, health-related costs, and physical activity/inactivity		✓	✓	✓
**Anthropometry**					
	Weight (in kg)		✓	✓	✓
	Height (in cm)		✓	✓	✓
	Waist circumference (in cm)	✓	✓	✓	✓
	Body fat (%)		✓	✓	✓
	Blood pressure		✓	✓	✓
	Indian Diabetes Risk Score	✓			
**Biochemical parameters**					
	Capillary blood glucose	✓			
	Venous fasting blood glucose		✓	✓	✓
	Postglucose load (2 hours)		✓		✓
	Fasting insulin assay		✓	✓	✓
	HbA1c		✓	✓	✓
	Lipid profile		✓	✓	✓

### Randomization

A study staff not involved in the study randomizes the participants into the intervention and control group across sites by the random allocation sequence method using a random numbers table. The randomization grid, which contained only the study unique ID number and inclusion criteria variables, is filled at each site by the site coordinators and sent to the personnel at the central randomization site as per the weekly randomization schedule. The grids are sent back to the sites post randomization. Therefore, the study personnel randomizing the participants are blinded to the group allocation of the participants, whereas the study team and participants at each site are not.

### Control Group

The control group participants receive usual care for patients with prediabetes or obesity at all the 3 study sites. They meet a nutritionist once after randomization and receive handouts reinforcing prevention of T2DM through increased physical activity and weight loss. All control group participants are offered access to the app at the end of the study.

### The Mobile App Description

An mHealth app called *mDiab* is used by all intervention group participants. mDiab has 12 weeks of video lessons that were created based on the Diabetes Community Lifestyle Improvement Program (D-CLIP) lessons and experiences [16], which in turn were originally developed using the US Diabetes Prevention Program. The video lessons are in the form of a reality television show, where real actors role-play and enact the concepts of lifestyle behavior change. This inspires and motivates the participants viewing the video lessons in the app to change their lifestyles to improve their health. For example, for the video lesson on incorporating a healthy diet, the actors act out ways to help the participant understand the concept and share experiences on the challenges faced while adopting it in real life. They also help in overcoming the challenges by suggesting suitable solutions. Apart from the video lessons, the app also has goal setting, alerts, and small text modules, which are brief write-ups on the video lessons. There are also multiple-choice questions to help reinforce learning. The 12 weeks of video lessons cover various aspects of T2DM prevention, with the goal of reducing diabetes risk through weight loss of at least 5%. The contents of the video lessons are described in Table 2 and Multimedia Appendix 2. Overall, the app has 3 important components—the video lessons as described above, tracking of behaviors, and weekly calls by the health coaches.

#### Tracking

**Figure 2:**
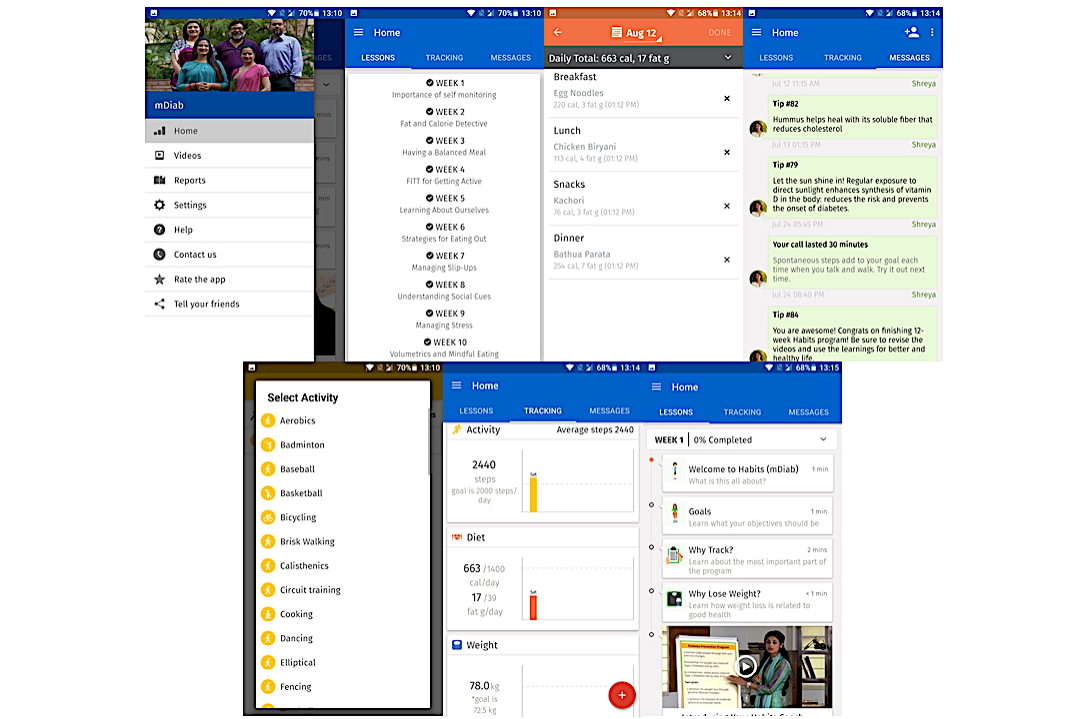
mDiab app screenshots.

This feature of the app is meant to enable users to track their diet, physical activity, and weight. The calorie and fat goals provided are based on the D-CLIP study. The participants are encouraged to track their daily food intake. The food database is developed using the Dr Mohan’s Diabetes Food Atlas [32] and National Institute of Nutrition’s guidelines [33]. The activity tracker automatically counts the number of steps using the mobile phone’s inbuilt accelerometer sensor. Additionally, the participant is encouraged to add any specific activity he/she likes to do. The weight tracker sets an initial weight loss goal of 5% and enables tracking. Every participant in the intervention group receives a weighing scale as an incentive to enable them to track their weight. The app sends out standardized reminders and motivational messages according to the progress of each user. Screenshots of the app’s tracking features are shown in Figure 2 for better understanding.

#### Communication With the Health Coach

Health coaches in the mDiab program are trained nutritionists who call participants once a week to inform them about the objectives of the video lessons and their progress and finally email them with a progress report using the data tracked by the respective participant. The participants also have the opportunity to communicate with the health coaches in any case via a message tool inbuilt in the app, and the health coaches respond to the participants in 24 hours.

### Procedures

#### Data Collection

At screening, the participant’s basic demographic details, IDRS, and CBG are measured. Post that, the study has a total of 3 testing time points including baseline, post intervention, and end of follow-up. All testing visits (except screening) occur after an overnight fast of at least 8 to 10 hours. The study participants are administered a questionnaire to record their sociodemographic data at baseline. Changes in diet and frequency of consumption of common foods are assessed using a 3-day diet recall, and short validated food frequency questionnaires are administered at all visits. Mobile phone usage and mHealth efficacy are also assessed at all visits. Physical activity and exercise behaviors are recorded using the short Madras Diabetes Research Foundation Physical Activity Questionnaire (MPAQ). MPAQ is a reproducible and validated instrument that captures data from multiple activity domains (including sedentary activity) over a period of a year from adults of both genders and varying ages from various walks of life, residing in India [34]. The participant’s QOL is assessed using the WHOQOL-BREF instrument comprising 26 items [35]. It measures 4 broad domains: physical health, psychological health, social relationships, and environment. We also aim to assess the cost-effectiveness of using the mHealth intervention by assessing the incremental costs and benefits per quality-adjusted life-year (QALY). The cost-effectiveness of the mHealth intervention from an individual and collective perspective is compared with usual care by conducting incremental cost-effective analyses in which the net costs and net effectiveness of the mHealth intervention and the usual care are calculated and expressed as a ratio. All analyses are within the time frame of the trial. The direct medical and nonmedical costs/indirect costs associated with the mHealth intervention over 10 to 12 months are included in the cost-effectiveness [36]. Program adherence is measured by monitoring how well the participants used the various features of the app by responding to reminders and alerts. This is tracked using a Web-based dashboard. A 75-g glucose tolerance test [37] at 0 and 120 min along with blood tests for insulin, glycated hemoglobin (HbA1c), and lipids are conducted at baseline and at the annual follow-up visit.

**Table 2:** *mDiab* overview of video lessons.

Sessions (Weekly)	Objectives
Session 1: Importance of self-monitoring	Effective and daily tracking using the habits program
	The importance of breakfast as a wholesome and balanced meal, along with sample meal plans
	How one can be in charge of their health beyond all daily challenges, including overloaded work schedule, trials of family life, involved social life, and healthy lifestyle changes
	Plan one’s day by planning the right time for eating
	Being true to oneself and achieving success
	Sleeping well for a healthy lifestyle
	Staying on track during the weekend
	Rewarding oneself
Session 2: Fat and calorie detective	Learning how fat and calories can affect health
	Learning where to find them in food
	Importance of portion sizes and measurement of food
	Being aware of fattening foods and hidden fats and switching to low-fat options to improve health and alleviate the risks of heart disease and diabetes
	Practicing being more aware and choosing food items consciously with lesser calories and fats, dwelling more into measuring foods
	Understanding that correlating the quantity of food eaten to its nutrient contribution (calories and fats) helps to remain within the fat and calorie limit for the day that is predetermined by one’s initial weight
	Using one’s skills to ration portions and choose food items judiciously by evaluating the consolidated caloric intake for the day; trying to find a balance through food and exercise
	Incorporating physical activity into your lifestyle
Session 3: Having a balanced meal	Understanding the importance of taking a balanced meal and learning what it looks like
	Getting the relevance of the “My plate” concept
	Understanding what it is to eat from different food groups
	Learning the good side of everything, especially fats
	Understand the balance between calories in and calories out by tipping the calorie balance and understanding the food serving sizes
Session 4: Being active	Techniques to make physical activity fun
	The FITT principle to balance your activity
	Exercises—leg raises and back extensions
	Reviewing what was learnt and putting it to practice
	Understanding the serious threat of sedentary lifestyle and keeping a track of F—frequency, I—intensity, T—type of activity, and T—time
Session 5: Learning about ourselves	Keeping food and activity cues by simplifying them to one’s core problems
	Learning how our environment causes us to be unhealthy
	Becoming aware of temptations that might steer one off course
	Steps to problem-solving
	Singling out areas in need of improvement and creating an action plan focusing on making these changes gradually
	Use tracking to one’s advantage
Session 6: Strategies for eating out	The healthy side of eating out
	Learning how to control what you eat when not at home
	Ordering healthy
	Planning ahead when going out to eat with friends or family
Session 7: Managing slip-ups	Managing and dealing with slip-ups
	Identifying the reasons for a slip-up
	Identifying negative thoughts and learning how to manage them
	Stretching exercises and learning some seated stretching techniques
	Understanding common external triggers for mismanaging diet and exercise and dealing with them
Session 8: Understanding social cues	Understanding social cues and how they affect us
	Making lifestyle changes using social cues to one’s advantage
	Being aware of your social interactions and how they affect you
	Learning to positively affect outcomes of unhealthy social cues
Session 9: Improving strength and flexibility	Improving strength and flexibility
	Strengthening one’s exercise program and learning resistance training
	Standing up for your health
	Incorporating strength training into your activity routine
Session 10: Volumetrics and eating mindfully	Understanding the importance and concept of volumetrics and eating mindfully
	High volume, low calorie foods—learning to eat more food that has fewer calories
	Eating mindfully by perceiving your physical and mental state
	Thinking before eating and being aware how one eats
	Paying attention to size, smell, texture, taste, and its other qualities
	Enjoying one’s meal to its fullest
Session 11: Stress management and staying motivated	Maintaining the momentum
	Recognizing positive lifestyle changes made so far
	Stress management
	Combating stress with planned activity or a healthy session of yoga
Session 12: Long-term heart health	Nurturing your heart into a healthy heart
	Understanding the importance of reducing risk of heart diseases by adopting positive lifestyle changes
	Understanding the importance of the new habits you have developed Using your skills to successfully keep the new habits you have created
	Keeping a schedule—tracking your new skill

At the post-intervention visit (3rd-month visit), fasting glucose, insulin, HbA1c, and lipids are measured. Height is measured at baseline visit, and anthropometry (height, weight, waist circumference, and body fat) and clinical measures (blood pressure) are measured at baseline, post intervention, and end of follow-up visits.

#### Program Acceptability

Program acceptability is assessed through focus group discussions (FGDs) with the intervention group participants at the end of follow-up. We plan to organize 2 FGDs at each site consisting of at least eight participants each. Thus, a total of 6 FGDs will be conducted in the study. The participants will be encouraged to share their experience on the benefits and problems of the intervention, financial worth of the program, and suggestions, if any, for improvement. Participants who drop out will also be contacted to understand their reasons for dropping out and to obtain their feedback.

#### Sample Size Calculation

Our study randomizes 1500 individuals across 3 sites to receive either the mHealth diabetes prevention program or usual care with a 6-month maintenance period. Considering an obesity prevalence of 20% as reported in the Indian Council of Medical Research-India Diabetes (ICMR-INDIAB) study [38,39] and with south Asians having a low BMI threshold compared with the rest of the population, 5% weight loss was determined as the primary outcome in the intervention group. On the basis of this assumption, a sample size of 588 individuals in each arm will be required to obtain a minimum of 3% difference in weight loss between groups with a power of 80% and a significance level of 5%. As this is a mobile phone–based intervention study, expecting a slightly higher dropout rate of 25%, 735 individuals in each group will need to be tested in the trial, resulting in a total of 1470, which has been rounded off to a total of 1500 individuals across 3 sites (500 per site).

All sample size and power calculations were done using the OpenEpi (The OpenEpi Project, Version 3, Atlanta, Georgia).

### Ethics Approval

The study protocol was approved by the Institutional Ethics Committee at Madras Diabetes Research Foundation, All India Institute of Medical Sciences, and the Human Research Ethics Committee at Deakin University, Australia. Consent is obtained from all participants. At baseline testing, all participants were also given a plain language statement of the study.

#### Statistical Analysis

Quantitative analyses of the obtained data will be conducted in STATA (StataCorp, Version 15, College Station, Texas) and SPSS (IBM SPSS Statistics for Windows, Version 24.0. Armonk, NY). A probability of <0.05 will be considered statistically significant for all tests. Effectiveness of the intervention will be assessed by measuring the differences in the study arms in terms of the primary outcome, weight loss and secondary outcomes, behavioral and social variables, and cost-effectiveness. Continuous variables will be assessed for normality, and anomalous values will be categorized. All variables will undergo descriptive analyses. *t* tests will be used to compare between the 2 groups. Data will be presented before and after adjusting for confounders. Qualitative data include analyses of the audiotaped transcripts from the FGDs. The transcripts will be de-identified, and textual data will be extracted from the same. Following this, key themes will be identified and reported.

#### Cost-Effectiveness

Two sets of outcomes are used to measure cost-effectiveness: cost-effectiveness analysis, which is the incremental cost per case of diabetes prevented, and cost-utility analysis, which is the incremental cost per QALY. The cost-effectiveness of the mHealth intervention is represented by the ratio of incremental cost to increment effectiveness. In addition, sensitivity analyses will be performed to examine effects of key parameters on cost-effectiveness ratios [36].

## Discussion

Mobile phone technology has been shown to have wide acceptance across various age groups and socioeconomic groups. It offers several opportunities for self-management as well as prevention of T2DM [40-42]. The mDiab study looks at the effect of combining various mHealth technologies that could all act as an effective tool for diabetes prevention in high-risk Asian Indians. The intervention is unique in that it is backed by evidence provided by the D-CLIP study [43]. The D-CLIP study was an RCT that compared an intervention group, which received 16 weekly sessions on lifestyle behavior change, nutrition, and physical activity, followed by 8 weeks of maintenance classes, with a control group that received standard of care. Individuals in the intervention arm who remained at high risk, that is, those with an HbA1c of >5.6% after 4 or more months in the program, received metformin, in addition to the lifestyle program. The goals for the individuals were set as weight loss of at least 7% and 150 min or more of moderate physical activity. In the mDiab intervention, however, we lowered the weight loss goal based on the learnings from the D-CLIP study as most Indians are not overtly obese. The D-CLIP results showed an almost 50% risk reduction in the obese population, and the numbers needed to treat were 6.8, which means that if 7 “high-risk cases” are given the intervention, one case of diabetes can be prevented [43].

On the basis of the D-CLIP results, the need to scale up diabetes prevention in India was felt, and mobile technology was thought of as a tool to introduce the intervention. This was the basis for the mDiab trial. Very few studies have been carried out in India to test the efficacy of mHealth technology in diabetes prevention. Ramachandran et al [44] studied the effect of SMS text messaging (short message service, SMS) in a high-risk population and showed that it is possible to decrease the incidence of diabetes with almost 10% difference between the groups by using a structured SMS text messaging intervention. However, their population sample was limited to working Indian men only. In another study that evaluated the impact of mobile SMS text messages on self-reported diabetes awareness and prevention behaviors among cell phone users in India, it was observed that the intervention group showed 15% improvement in their dietary and physical activity behaviors compared with the control group [45].

Globally, many studies have shown significant improvements in glycemic control using mobile phone technology. Hussein et al [46] showed that continuous and individualized support and interaction with a diabetes educator and a clinician through SMS text messages could decrease the HbA1c by 1.16% in individuals with type 2 diabetes. Studies using SMS technology, based on knowledge, attitude, practice, and self-efficacy and motivation, have shown improvement in glycemic control [47,48]. Some studies have also looked at secondary prevention of diabetes by enforcing self-management of lifestyle behavior, insulin therapy, medications, and physician visits [49,50]. The study by Browne et al [51] reported that younger adults are more susceptible to using eHealth services for diabetes self-management. This may be because younger adults were tech-savvy and found using eHealth self-management supports time-saving. The Diabetes MILES (Management and Impact for Long-term Empowerment and Success)—Australia study [52] reported that telehealth and eHealth are promising areas for diabetes management, especially to reach out to wider population groups.

In the context of rapid adoption of mobile technology [18] and the fast rise of the T2DM epidemic in LMICs such as India [1-3], mDiab is an RCT testing the effectiveness, cost-effectiveness, and sustainability of a culturally tailored mHealth diabetes prevention program for Asian Indians. If successful, mDiab can be used as a model for translational and implementation research on the use of mHealth technology for diabetes prevention. Indeed, the mDiab experience can be further expanded to the prevention of other noncommunicable diseases such as hypertension and cardiovascular diseases.

## Appendix

Multimedia Appendix 1 Summary of study measures.

Multimedia Appendix 2 “mDiab” overview of video lessons.

## Abbreviations

BMI: body mass index
CBG: capillary blood glucose
D-CLIP: Diabetes Community Lifestyle Improvement Program
FGDs: focus group discussions
IAMAI: Internet and Mobile Association of India
ICMR-INDIAB: Indian Council of Medical Research-India Diabetes
IDRS: Indian Diabetes Risk Score
IFG: impaired fasting glucose
IGT: impaired glucose tolerance
IMRB: Indian Market Research Bureau
LMICs: low- and middle-income countries
mHealth: mobile health
MPAQ: Madras Diabetes Research Foundation Physical Activity Questionnaire
QALY: quality-adjusted life-year
QOL: quality of life
RCT: randomized controlled trial
SMS: short message service
T2DM: type 2 diabetes mellitus
WHO: World Health Organization
