# Supplementary material for: Mobile Health Technology (mDiab) for the Prevention of Type 2 Diabetes: Protocol for a Randomized Controlled Trial
Source: JMIR Res Protoc. 2017 Dec 12;6(12):e242. doi: 10.2196/resprot.8644 (PMC5743924; doi:10.2196/resprot.8644)
Supplement: Multimedia Appendix 1 [file resprot_v6i12e242_app1.pdf]

TABLE 1: Summary of study measures				
**MEASUREMENTS**	**Screening**	**Baseline**	**Post-intervention**	**End of follow-up**
**Questionnaires**				
Short questionnaire	✓			
Questionnaires measuring participants' health habits, diet behavior, quality of life, health related costs and physical activity/inactivity		✓	✓	✓
**Anthropometry**				
Weight (kg)		✓	✓	✓
Height (cm)		✓	✓	✓
Waist circumference (cm)	✓	✓	✓	✓
Body fat (%)		✓	✓	✓
Blood pressure		✓	✓	✓
Indian Diabetes Risk Score	✓			
**Biochemical parameters**				
Capillary blood glucose	✓			
Venous fasting blood glucose		✓	✓	✓
Post glucose load (2 hour)		✓		✓
Fasting insulin assay		✓	✓	✓
Glycated hemoglobin (HbA1c)		✓	✓	✓
Lipid profile		✓	✓	✓
