# Supplementary material for: Mobile Health Technology (mDiab) for the Prevention of Type 2 Diabetes: Protocol for a Randomized Controlled Trial
Source: JMIR Res Protoc. 2017 Dec 12;6(12):e242. doi: 10.2196/resprot.8644 (PMC5743924; doi:10.2196/resprot.8644)
Supplement: Multimedia Appendix 2 [file resprot_v6i12e242_app2.pdf]

TABLE 2: “mDiab” overview of video lessons

SESSIONS (WEEKLY)	OBJECTIVES
Session 1: Importance of self-monitoring	• Effective and daily tracking using the habits program<br>• The importance of breakfast as a wholesome and balanced meal, along with sample meal plans<br>• How one can be in-charge of their health beyond all daily challenges including overloaded work schedule, trials of family life, involved social life, healthy lifestyle changes<br>• Plan one’s day by planning the right time for eating<br>• Being true to oneself and achieving success<br>• Sleeping well for a healthy lifestyle<br>• Staying on track during the weekend<br>• Rewarding oneself
Session 2: Fat and calorie detective	• Learning how fat and calories can affect health<br>• Learning where to find them in food<br>• Importance of portion sizes and measurement of food<br>• Being aware of fattening foods and hidden fats and switching to low fat options to improve health and alleviate the risks of heart disease and diabetes

	• Practicing being more aware and choosing food items consciously with lesser calories and fats, dwelling more into measuring foods<br>• Understanding that correlating the quantity of food eaten to its nutrient contribution (calories and fats) helps to remain within the fat and calorie budget for the day that is predetermined by one's initial weight<br>• Using one's skills to ration portions and choose food items judiciously by evaluating the consolidated caloric intake for the day. Trying to find a balance through food and exercise.<br>• Incorporating physical activity into your lifestyle.
Session 3: Having a balanced meal	• Understanding the importance of taking a balanced meal and learning what it looks like<br>• Getting the relevance of the "My plate" concept<br>• Understanding what it is to eat from different food groups<br>• Learning the good side of everything especially fats<br>• Understand the balance between calories in and calories out by tipping the calorie balance and understanding the food serving sizes
Session 4: Being active	• Techniques to make physical activity fun<br>• The FITT principle to balance your activity

	• Exercises - Leg raises and back extensions<br>• Reviewing what was learnt and putting it to practice<br>• Understanding the serious threat of sedentary lifestyle and keeping track of,<br>- F – frequency<br>- I – intensity<br>- T – type of activity<br>- T – time
Session 5: Learning about ourselves	• Keeping food and activity cues by simplifying them to one's core problems<br>• Learning how our environment causes us to be unhealthy<br>• Becoming aware of temptations that might steer one off course<br>• Steps to problem solving<br>• Singling out areas in need of improvement and creating an action plan focusing on making these changes gradually<br>• Use tracking to one's advantage
Session 6: Strategies for eating out	• The healthy side of eating out<br>• Learning how to control what you eat when not at home<br>• Ordering healthy<br>• Planning ahead when going out to eat with friends or family
Session 7: Managing slip-ups	• Managing and dealing with slip ups<br>• Identify the reasons for a slip up

	• Identifying negative thoughts and learning how to manage them<br>• Stretching exercises and learning some seated stretching techniques.<br>• Understanding common external triggers for mismanaging diet and exercise and dealing with them
Session 8: Understanding social cues	• Understanding social cues and how they affect us<br>• Making lifestyle changes using social cues to one's advantage<br>• Being aware of your social interactions and how they affect you<br>• Learning to positively affect outcomes of unhealthy social cues
Session 9: Improving strength and flexibility	• Improving strength and flexibility<br>• Strengthening one's exercise program and learning resistance training<br>• Standing up for your health<br>• Incorporating strength training into your activity routine
Session 10: Volumetrics and eating mindfully	• Understanding the importance and concept of volumetrics and eating mindfully<br>• High volume, low calorie foods - Learning to eat more food that has fewer calories<br>• Eating mindfully by perceiving your physical and mental state<br>• Thinking before eating & being aware how one eats<br>• Paying attention to size, smell, texture, taste and its other qualities<br>• Enjoying your meal to its fullest

Session 11: Stress management and staying motivated	• Maintaining the momentum<br>• Recognizing positive lifestyle changes made so far<br>• Stress management<br>• Combating stress with planned activity or a healthy session of yoga
Session 12: Long-term heart health	• Nurturing your heart into a healthy heart<br>• Understanding the importance of reducing risk of heart diseases by adopting positive lifestyle changes<br>• Understanding the importance of the new Habits you have developed<br>• Using your skills to successfully keep the new habits you have created<br>• Keeping a schedule – tracking, your new skill
